# Kinetics of human myeloid-derived suppressor cells after blood draw

**DOI:** 10.1186/s12967-015-0755-y

**Published:** 2016-01-06

**Authors:** Eva Grützner, Renate Stirner, Lukas Arenz, Anastasia P. Athanasoulia, Kathrin Schrödl, Carola Berking, Johannes R. Bogner, Rika Draenert

**Affiliations:** Sektion Klinische Infektiologie, Medizinische Klinik und Poliklinik IV, Klinikum der Universität München, Pettenkoferstr. 8a, 80336 Munich, Germany; Medizinische Klinik und Poliklinik V, Klinikum der Universität München, Ziemssenstr. 1, 80336 Munich, Germany; Klinik und Poliklinik für Dermatologie und Allergologie, Frauenlobstraße 9–11, 80337 Munich, Germany

**Keywords:** Myeloid-derived suppressor cells (MDSC), MDSC subsets, MDSC, Kinetics, Blood processing

## Abstract

**Background:**

Human myeloid-derived suppressor cells (MDSC) have been described as a group of immature myeloid cells which exert immunosuppressive action by inhibiting function of T lymphocytes. While there is a huge scientific interest to study these cells in multiple human diseases, the methodological approach varies substantially between published studies. This is problematic as human MDSC seem to be a sensible cell type concerning not only cryopreservation but also time point after blood draw. To date data on delayed blood processing influencing cell numbers and phenotype is missing. We therefore evaluated the kinetics of granulocytic MDSC (gMDSC) and monocytic MDSC (mMDSC) frequencies after blood draw in order to determine the best time point for analysis of this recently defined cell type.

**Methods:**

In this study, we isolated peripheral blood mononuclear cells (PBMC) of patients with HIV infection or solid tumors directly after blood draw. We then analyzed the frequencies of gMDSC and mMDSC 2, 4 and 6 h after blood draw and after an overnight rest by FACS analysis using the standard phenotypic markers. In addition, part of the cells was frozen directly after PBMC preparation and was measured after thawing.

**Results:**

gMDSC levels showed no significant difference using fresh PBMC over time with a limitation for the overnight sample. However they were massively diminished after freezing (p = 0.0001 for all subjects). In contrast, frequencies of fresh mMDSC varied over time with no difference between time point 2 and 4 h but a significantly reduction after 6 h and overnight rest (p = 0.0005 and p = 0.005 respectively). Freezing of PBMC decreased the yield of mMDSC reaching statistical significance (p = 0.04). For both MDSC subgroups, FACS analysis became more difficult over time due to less sharp divisions between populations.

**Conclusions:**

According to our data human MDSC need to be studied on fresh PBMC. gMDSC can be studied with delay, mMDSC however should be studied no later than 4 h after blood draw. These results are crucial as an increasing number of clinical trials aim at analyzing MDSC nowadays and the logistics of blood processing implies delayed sample processing in some cases.

**Electronic supplementary material:**

The online version of this article (doi:10.1186/s12967-015-0755-y) contains supplementary material, which is available to authorized users.

## Background

In the last decades newly described suppressors of the cellular immune system have become increasingly important: myeloid derived suppressor cells (MDSC). These cells are part of a group of immature cells with myeloid origin which act immunosuppressively by inhibiting function of T lymphocytes among others [1]. Firstly discovered in mice, MDSC have been shown in various studies in patients with solid tumors [2] like hepatocellular carcinoma (HCC) [3], non-small cell lung cancer (NSCLC) [4], head and neck cancer [4], gastrointestinal cancer [5] or melanoma [6, 7]. Recently MDSC were also found in peripheral blood of infectious diseases namely HIV [8] or tuberculosis [9]. All these studies on human MDSC vary substantially in their methodological approach. For this reason, translational studies on human MDSC are difficult to compare especially regarding two criteria: phenotypic markers and time point of analysis after blood draw.

MDSC can be divided in two main subgroups: granulocytic MDSC (gMDSC) and monocytic MDSC (mMDSC) [1]. The markers to characterize human MDSC are heterogeneous and a lot of different markers are used depending on tumor or infection [1, 2, 8]. In studies in infectious diseases (HIV, HCV), gMDSC were defined to lack the expression of CD14 but to express CD15/CD33/CD11b [8, 10] whereas mMDSC were characterized as CD14^+^/CD11b^+^/HLA-DR^−/low^ cells [8, 10, 11] or additionally CD33^+^ cells [12, 13]. Other marker combinations are CD33^high^/CD66b^high^/IL-4Rα^inter^/HLADR^dim^ for *Pseudomonas aeruginosa* infection in cystic fibrosis [14] or LIN^−/low^/HLA-DR^−/low^/CD33^+^/CD11b^+^ for tuberculosis [9]. Previously mentioned studies in cancer characterized gMDSC as CD33^+^/HLA-DR^−^/CD66b^+^ [4] and mMDSC as CD14^+^ and HLA-DR^−/low^ [3, 5–7]. The usage of a lineage cocktail [9, 15] is less frequent in recent studies. Dumitru et al. postulated a marker combination of CD11b/CD14/CD66b/CD33/HLA-DR/CD16 for identification of gMDSC and mMDSC within one peripheral blood mononuclear cells (PBMC) sample [2]. In conclusion, to date the most established markers for gMDSC are CD14^−^, CD33^+^, CD66b^+^/CD15^+^ and CD11b^+^ and for mMDSC CD33^+^, CD14^+^ and HLA-DR^−/low^.

Other reasons which complicates the comparison of studies are the cryopreservation or kinetics of MDSC frequencies after blood draw which seem to have an immense influence on the results. So far, there is data on the effect of cryopreservation of MDSC. Trellakis et al., Kotsakis et al. and Duffy et al. [5, 15, 16] showed that freezing/thawing procedures of PBMC had influence on frequencies of MDSC and also on their function [15]. However, Trellakis et al. studied mainly gMDSC. Kotsakis et al. used phenotypic markers that are disparate to the markers used today as mentioned above rendering the data not transferrable to MDSC subsets used today. In spite of the existing data, many studies still use frozen PBMC to evaluate MDSC frequencies. Therefore the harmful effects of cryopreservation on MDSC cannot be stressed enough.

Besides the influence of freezing PBMC, data on the time point of PBMC processing after blood draw is missing. In clinical settings, several hours often pass until the study subjects’ blood can be processed not only for observational studies but also in clinical trials. Looking at a sensible cell type, it is highly important to evaluate the rate of decay of these cells with increasing time after blood draw.

The aim of this study was to standardize the analytical process for assessing human MDSC. As critical aspects, we analyzed the time frame between blood draw and cell analysis and the recovery rate after freezing of gMDSC and mMDSC defined by standard phenotypic markers. Our results show that gMDSC can be used freshly after delayed processing with a reservation towards the overnight rest. We confirm that they cannot be analyzed after cryopreservation. In contrast, we show that fresh mMDSC are significantly lost when rested for more than four hours after blood draw. However the recovery rate after freezing was better for this cell subtype.

## Methods

### Study subjects

42 individuals participated in the study after signing informed consent. The study was approved by the Institutional Review Board of the Ludwig-Maximilians-Universität, Munich, Germany.

The study subjects were divided into the following groups: 25 patients with chronic viral infection [HIV-infected patients with no or less than 4 weeks of antiretroviral therapy (CD4 counts: median 165/µl, range 1–891/µl; viral loads: median 37,350 copies/ml, range <50–3,365,672 copies/ml): 24; HCV-infected patient (Genotype 1b; viral load: 240,000 I.U./ml): 1], and 17 subjects with advanced stage solid tumors before specific treatment (malignant melanoma: 9; non-small cell lung carcinoma (NSCLC): 6; hepatocellular carcinoma (HCC): 1; tracheal tumor: 1).

### PBMC isolation, freezing and thawing

PBMC were isolated from freshly obtained EDTA blood by Ficoll density gradient centrifugation (Biocoll Separation Solution, Biochrom, Germany). Sample processing was performed 2, 4, 6 h and 20–26 h (= overnight) after blood draw. Between time points starting at approximately 2 h after blood draw, PBMC were stored in RPMI 1640 (10 ml; PAA, Austria or Biochrom, Germany) supplemented with 10 % FCS (Biochrom, Berlin, Germany), 1 % penicillin–streptomycin (Biochrom, Germany), 1 % L-Glutamin (PAA, Austria) and 1 % Hepes (Sigma-Aldrich, Germany) at 37 °C and 5.0 % CO_2_. Samples were not stored as whole blood sample.

For freezing, PBMC (2 h after blood draw and directly after PBMC preparation) and freezing medium [90 % fecal calf serum (FCS) + 10 % DMSO] were pre-chilled on ice [16]. PBMC were resuspended in 1 ml freezing medium. The cryovial was transferred into a pre-chilled Nalgene™ Cryo 1 °C freezing container (cooling rate −1 °C/min; Nalgene^®^, USA) and stored at −80 °C overnight. For long-term storage, vials were transferred into a liquid nitrogen storage container the next day.

PBMC were thawed by warming up frozen vials at 37 °C in a water bath. PBMC were then transferred into supplemented RPMI 1640 (as described above) which was pre-chilled on ice and washed twice in media. The viability of cells was determined by trypan blue dye exclusion method and was more than 88 %. Cells were used for assays immediately after thawing.

### Flow cytometric analysis

Extracellular staining with fluorescent antibodies on fresh or frozen PBMC was done as described previously [8]. At each time point, analyses were performed for gMDSC and mMDSC respectively. The following antibodies were used: gMDSC: CD11b-FITC, CD14-APC, CD15-PerCP, CD66b-PE [14, 17]; mMDSC: CD11b-FITC, CD14-APC, CD33-PE, HLA-DR-PerCP, PerCP isotype control (all BioLegend, USA). Cells were analyzed on a FACSCalibur (BD, Germany) and analysis of data was done using FlowJo software 7.2.1 (TreeStar, Inc. Ashland, OR). Gating strategies were according to Vollbrecht et al. [8] and Rieber et al. [14, 17] for gMDSC. For the gating of mMDSC, we used a PerCP isotype control in order to gate for HLA-DR and our gating strategy was according to Dumitru et al. [2] (see Additional file 1: Figure S1). For the statistics, we indicated MDSC as percentage of PBMC in all subgroups because the numbers of monocytes vary substantially in subjects with e.g. chronic viral infections. However, in the dot plots shown in Fig. 2, frequencies of the described populations are shown in percentages of monocytes.

### Statistical analysis

Statistical analysis was performed using GraphPad Prism version 5.0. Differences between groups were determined by Mann–Whitney test. A p value of <0.05 was considered to be statistically significant.

## Results

### No significant difference in frequencies of gMDSC after delayed sample processing, but significant reduction of mMDSC frequencies after 6 h and overnight rest

**Fig. 1:**
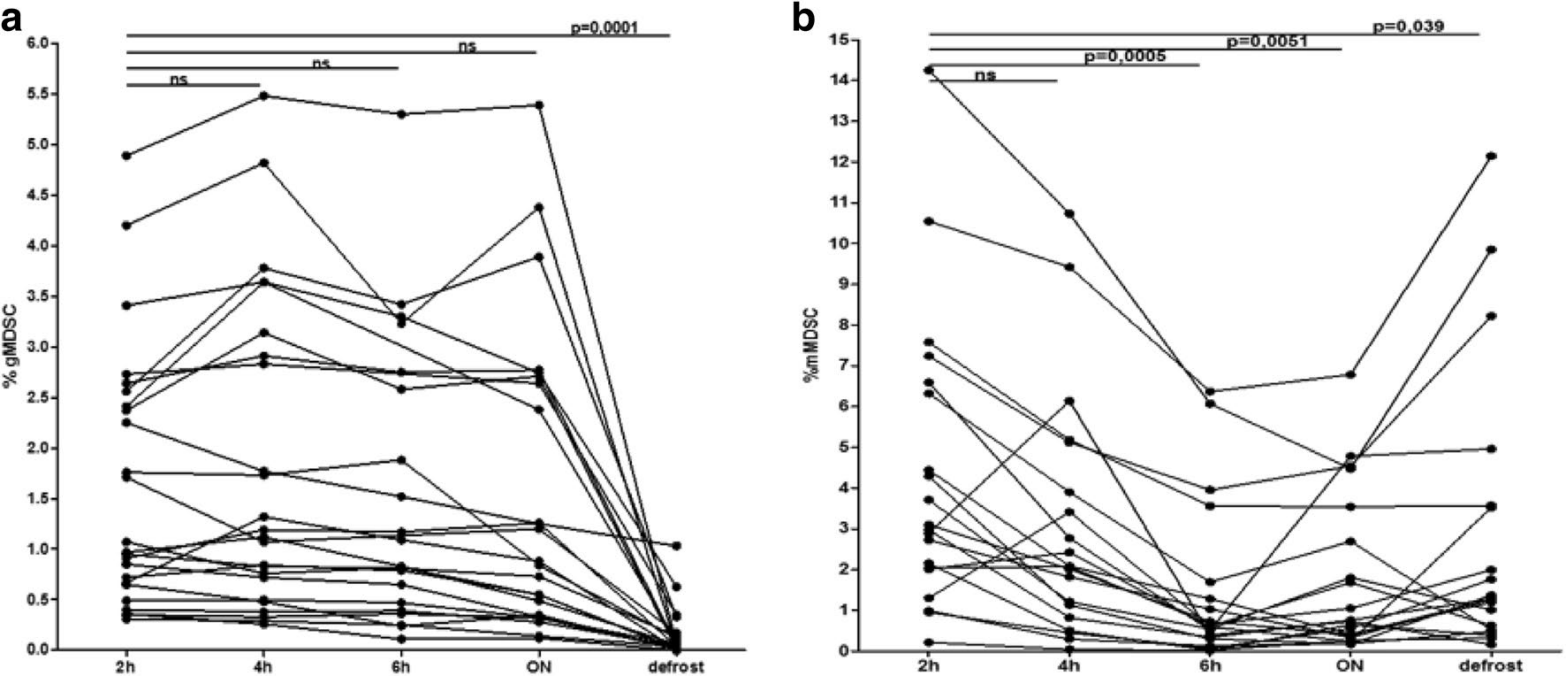
Kinetics of gMDSC and mMDSC levels. Frequency of gMDSC (**a**) and mMDSC (**b**) within PBMC after 2, 4, 6 h, overnight rest (ON) and freezing/thawing procedure (defrost). PBMC were stored in RPMI media after the ficoll separation. gMDSC were analysed in 24 patients with chronic viral infections and mMDSC in 17 patients with solid tumors and three HIV infected patients. There was no statistical significant difference in gMDSC frequencies over time (2, 4, 6 h, ON). Statistical significant reduction of gMDSC frequencies after freezing/thawing procedure (p = 0.0001; Mann–Whitney test). The difference in frequencies in mMDSC is not significant after 4 h whereas their numbers were significantly reduced after a 6 h and overnight rest (p = 0.0005 and p = 0.005, respectively; Mann–Whitney test). mMDSC were more stable towards freezing/thawing, however the difference reached statistical significance (p = 0.039; Mann–Whitney test)

In comparison to T lymphocytes which can be frozen or cultured over weeks without altering their phenotype [18–23], MDSC are a rather sensible cell type. In order to determine the best time point for MDSC analysis after blood draw, we first studied the kinetics of gMDSC frequencies. Frequencies of CD11b^+^/CD14^−^/CD15^+^/CD66b^+^ gMDSC after 2 h compared to 4, 6 h and even after an overnight rest were not significantly different in 24 patients with chronic viral infections (p > 0.5) (Fig. 1a). After cryopreservation, the frequencies of gMDSC dropped to under 0.4 % in 22 of the patients, independently of the frequencies at 2 h, and were reduced by 92 % on average. The loss of gMDSC numbers after freezing/thawing was statistically highly significant (p = 0.0001).

**Fig. 2:**
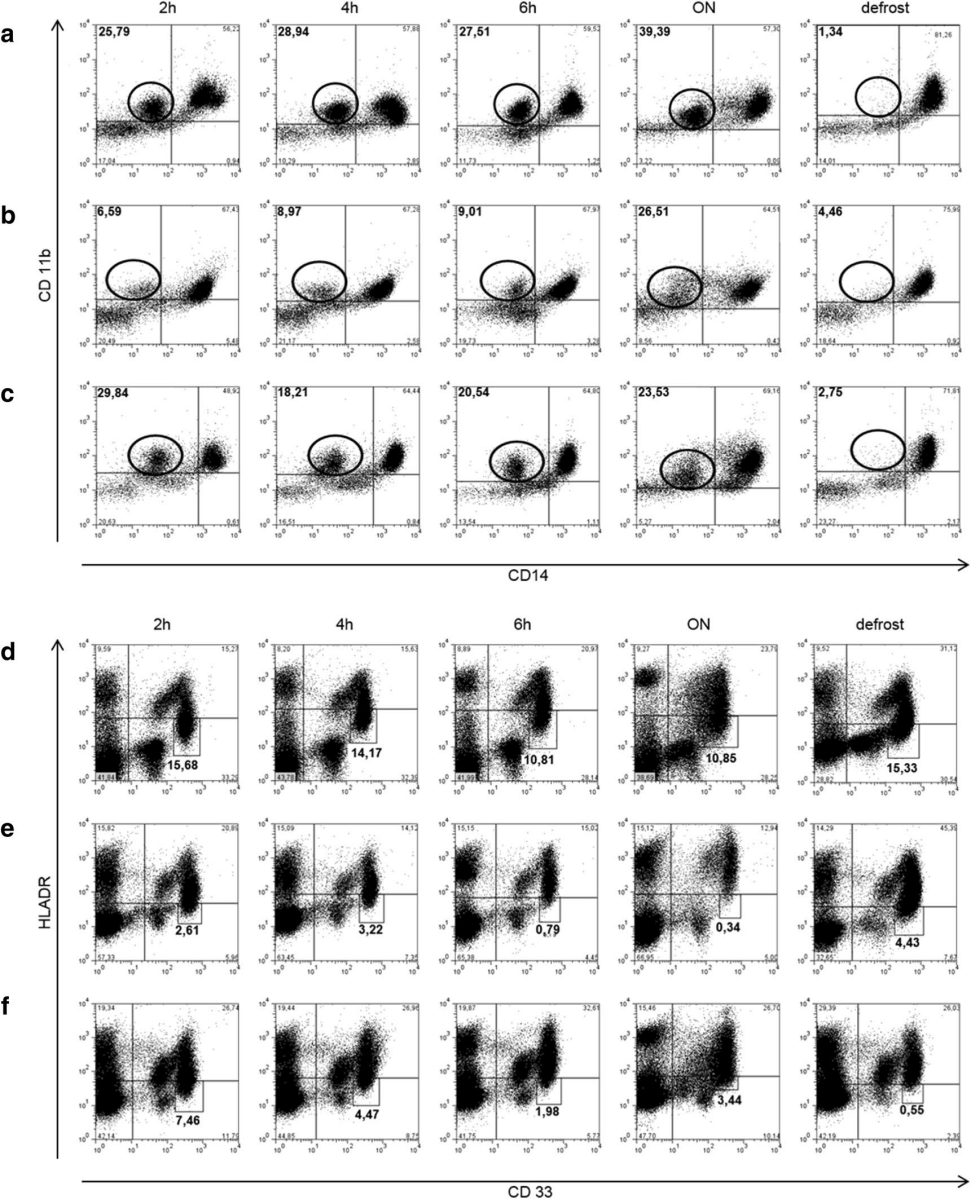
Dot blot kinetics of MDSC. Representative dot plots of gMDSC (**a–c**) and mMDSC (**d–f**) after 2, 4, 6 h, overnight rest and freezing/thawing procedure of three HIV patients (gMDSC) and three patients with solid tumors (mMDSC). PBMC were stored in RPMI media after the ficoll separation gMDSC gated on CD11b^+^ and CD14^−^ population (*oval* gate); for CD15^+^/CD66b^+^ gating see Additional file 2: Figure S2; mMDSC gated on CD33^+^ and HLA-DR^low/−^ (*rectangle* gate); CD14^+^ gating not shown. The majority of dot blots (**a**) of the CD11b^+^/CD14^−^ population remained similar after 2, 4 and 6 h. However, it was not clearly definable after ON and almost disappeared after the freezing/thawing procedure. Divergent minor presentations were seen and are presented in the remaining panels: increasing gMDSC frequencies after overnight rest and relatively high gMDSC levels after freezing/thawing (**b**) as well as reduction of gMDSC numbers at 4, 6 h and overnight rest compared to the 2 h time point (**c**). Looking at mMDSC (CD33^+^ and HLADR^−/low^ population), again the majority of dot blots presented as shown in 2d: similar numbers of mMDSC after 2 and 4 h and a clear reduction in mMDSC frequencies after 6 h. Populations are not well discriminable after overnight rest and after freezing/thawing. Minor divergent presentations are shown in the remaining panels: very high mMDSC frequencies after freezing/thawing (**e**) as well as increased frequencies after overnight rest and loss of mMDSC after freezing/thawing (**f**)

For mMDSC, we used only three patients with HIV infection because mMDSC levels in the other patients with chronic viral infections were too low. In addition we recruited 17 study subjects with advanced stages of solid tumors (for entities see “Methods” section) who exhibited high mMDSC frequencies. Overall, we found comparable CD33^+^/CD14^+^/HLA-DR^−/low^ mMDSC frequencies after 2 and 4 h in the 20 individuals studied. However, we observed a remarkable drop in frequencies after 6 h and overnight rest by 75 and 47 % which was statistically highly significant (p = 0.0005 and p = 0.005, respectively). Compared to gMDSC, mMDSC were not as sensitive to the freezing/thawing procedure. For the defrost sample, the overall reduction averaged 30 %. Compared to 2 h, in 5 subjects the rates of mMDSC were even higher (Figs. 1b, 2e) and in 3 subjects they were almost equal (reduction less than 15 %). None the less, the difference between the 2 h time point and the freezing/thawing sample reached statistical significance (p = 0.04, Fig. 1b).

In conclusion, gMDSC can be processed with delay without changing the result significantly whereas mMDSC are sensitive to resting for hours and PBMC should be processed within 4 h after blood draw for mMDSC analysis. Our results confirm that gMDSC cannot be studied from frozen PBMC. mMDSC are less sensitive to freezing. According to our data, they should still not be studied in frozen samples.

### Alterations in MDSC phenotype over time

In the next step, we compared the dot blots of the various time points of gated MDSC as a shifting of the populations had been clearly visible on first inspection.

Based on the statistics, gMDSC can even be measured after an overnight rest. However comparison of the dot blots showed that the populations get more blurred over time (Fig. 2a–c). Especially in the overnight sample, the marker CD11b cannot be discriminated into positive and negative cells any more. This led to much higher numbers of gMDSC in a minority of samples (see Fig. 2b overnight time point). In addition we observed 5 individuals in whom gMDSC frequencies were substantially reduced after the 2 h time point (Fig. 2c). Figure 2a–c also shows well the loss of gMDSC after freezing/thawing. Dot blots of the CD66b^+^ and CD15^+^ population differed less after short term storage (see Additional file 2: Figure S2).

In analogy, we assessed the dot blots of mMDSC comparing the different time points. The most striking phenomenon was that the marker HLA-DR stretched to higher values over time (Fig. 2d–f). This necessitated an adjustment of the gating cut-off between HLA-DR^high^ and HLA-DR^low^. We gated HLA-DR using an isotype control because this marker does not separate in two distinct populations (i.e. HLA-DR positive and HLA-DR negative). A shifting of this population renders the marker—which is difficult to gate to begin with—even more challenging to gate.

As the PBMC were frozen directly after the ficoll separation (2 h time point), the dot blots of mMDSC of the freezing/thawing time point resembled those of the 2 h time point with regard to HLA-DR (Fig. 2d–f). In the overnight sample the cell populations again got blurred with respect to CD33 (Fig. 2d–f). CD14 was the most stable marker in this setting (data not shown). Interestingly, there were also 5 samples where mMDSC were almost lost (reduction more than 70 % in comparison to 2 h time point) after freezing/thawing (Fig. 2f).

These observations raise the reservation about resting PBMC overnight for gMDSC analysis and they underscore the conclusion that mMDSC should not be analyzed later than 4 h after blood draw.

## Discussion

Currently it is rarely feasible to compare the results of studies on human MDSC due to strongly differing approaches with regard to the preparation of the cells for analysis. Detailed information on time between blood draw and analysis of cells or their cryopreservation is often not provided in the respective publications [8, 13, 15, 24]. The knowledge of the influence of these variables on the cells is poor to date. In this study we analyzed a critical aspect in MDSC research, namely the impact of short time storage of PBMC before analyses of MDSC which, to our knowledge, has not been investigated to date. Our results show that gMDSC tolerate storage up to 26 h at 37 °C and 5.0 % CO_2_ with limitations, but are not viable after freezing. mMDSC however should not be stored for more than 4 h after blood draw. Freezing is better tolerated by mMDSC.

It has been shown that granulocytes in peripheral blood are sufficiently viable for up to 22–26 h after blood draw, however they do change their density over time [25]. gMDSC are closely related to granulocytes [2] and their resistance towards delayed analysis is comparable to granulocytes [25]. In addition, we also observed a markedly changing phenotype of gMDSC after overnight rest, especially as far as CD11b was concerned, less pronounced for CD14. It has even been shown that the change in density of granulocytes leads to a significant increase of contaminating granulocytes within PBMC when the ficoll separation is done after 22–26 h [25]. We can exclude this contamination in our experiments as the ficoll separation of the blood samples was done right after blood draw and only PBMC in RPMI were used for storage.

Time points later than 4 h after blood draw showed significant reductions in mMDSC frequencies. We assume this is due to the adhesion of the monocytic cells to the vial surface. Under physiological conditions monocytes which are related to mMDSC circulate in the bloodstream until they differentiate e.g. to macrophages in inflamed tissue. Therefore monocytes have to adhere and migrate through the vascular endothelium [26, 27]. In vitro the ability of monocytes to adhere to glass surfaces was first described in 1966 [28]. This adhesion was shown to be dependent on the surface material [29–32]. Interestingly, mMDSC frequencies were lowest at the 6 h time point and again higher after overnight rest which was not statistically significant. We cannot explain this phenomenon at the moment. We can only speculate that the monocytic cells release their attachment to the vial surface after some time.

Our analyses also showed that resting of cells increased the gating difficulties for mMDSC as well. The marker HLA-DR is difficult to gate in general and we used an isotype control to overcome this problem. Short term storage led to an additional blurring of the HLA-DR marker in the dot blots that was even challenging to overcome with the isotype control.

The data on freezing of gMDSC was impressive with an almost complete loss of the gMDSC population after thawing. This seems natural as gMDSC are closely related to granulocytes and granulocytes do not tolerate freezing [33–35]. It is in line with data by Trellakis et al. who showed a reduction of gMDSC frequencies of more than 50 % in all tested samples after freezing [16]. Another study on freezing/thawing of MDSC used a lineage cocktail rendering the comparison of their data with ours difficult. However the population closest to our gMDSC, namely HLA-DR^−^/LIN^−^/CD15^+^, also showed a 69 % reduction after freezing [15]. There is even less data on freezing/thawing of mMDSC. In our hands, mMDSC were more resistant to the freezing procedure. However the difference between the 2 h time point and the frozen sample still reached statistical significance. Published data is conflicting in this point: while one study stated similar sensitivity towards freezing comparing mMDSC and gMDSC [16], two studies found no significant difference between the frequency of fresh and frozen cells using the markers HLA-DR^−^/CD14^+^ for definition of cells. Duffy et al. even showed increased frequencies within frozen PBMC [5]. However, Kotsakis et al. reported abolished function of MDSC after freezing [15]. Consequently, based on our research and the literature, we cannot recommend using frozen samples for gMDSC or mMDSC analyses. If mMDSC have to be analyzed in frozen PBMC, ficoll separation and freezing should be done directly after blood draw as suggested by the results of the kinetics of mMDSC. In view of the reality of translational research with the clinical parts of studies being often conducted at different sites than the laboratory evaluation—sometimes even in different countries—this is unfortunate. However the data clearly shows that freezing of MDSC has to be avoided in order to accurately describe the situation in vivo.

## Conclusions

MDSC are a very sensitive type of cells of the immune system in terms of time point of analysis. Their precise role in cancer and infectious diseases still remains an urgent target for future investigations which should be facilitated by harmonizing the analytical process. Our data show differences in sensitivity of gMDSC and mMDSC and are crucial for defining the ideal time point for analysis. For frozen/thawed gMDSC and mMDSC, we support the results from previous studies to analyse fresh PBMC. Of particular importance is our finding determining the first 4 h after blood draw as ideal time point for analyses of mMDSC but allowing processing of gMDSC within the same day.

## Additional files

10.1186/s12967-015-0755-y Gating strategies for gMDSC and mMDSC.
10.1186/s12967-015-0755-y Gating of CD15^+^/CD66b^+^ population.

## Abbreviations

gMDSC: granulocytic myeloid-derived suppressor cells
HCC: hepatocellular carcinoma
MDSC: myeloid-derived suppressor cells
mMDSC: monocytic myeloid-derived suppressor cells
NSCLC: non small cell lung cancer
PBMC: peripheral blood mononuclear cells
