# Supplementary material for: Kinetics of human myeloid-derived suppressor cells after blood draw
Source: J Transl Med. 2016 Jan 6;14:2. doi: 10.1186/s12967-015-0755-y (PMC4702395; doi:10.1186/s12967-015-0755-y)
Supplement: Supplementary file 1 — 10.1186/s12967-015-0755-y Gating strategies for gMDSC and mMDSC. [file 12967_2015_755_MOESM1_ESM.docx]

**Figure S1: Gating strategies for gMDSC and mMDSC**

**gMDSC**

**
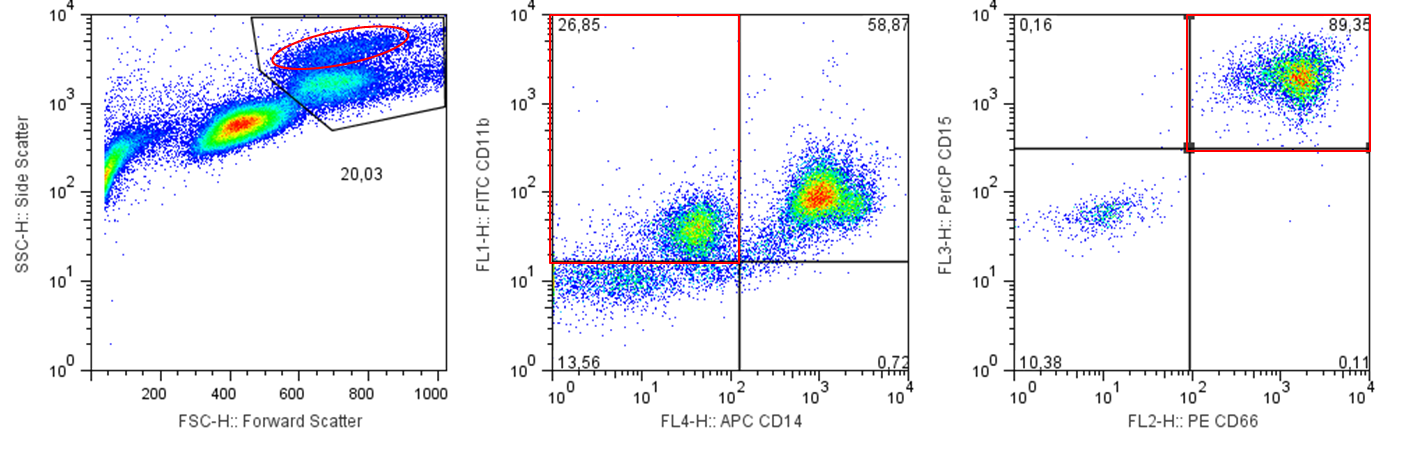
**

Representative dot blots and gating strategies for gMDSC. The first gate (black gate, first panel) is placed on the monocyte fraction in FSC and SSC. The main fraction of MDSC is found in the population above the monocyte fraction (oval red gate, first panel). Second gate shows the CD11b^+^ and CD14^-^ population (red square gate, second panel). In the third panel (red square gate, third panel) cells are CD11b^+^/CD14^-^ and CD66b^+^/CD15^+^ and are defined as gMDSC. Gating was performed according to Vollbrecht et al. (AIDS 2012) and Rieber et al. (J Immunol 2013, Clin Exp Immunol 2013).

**mMDSC**

**
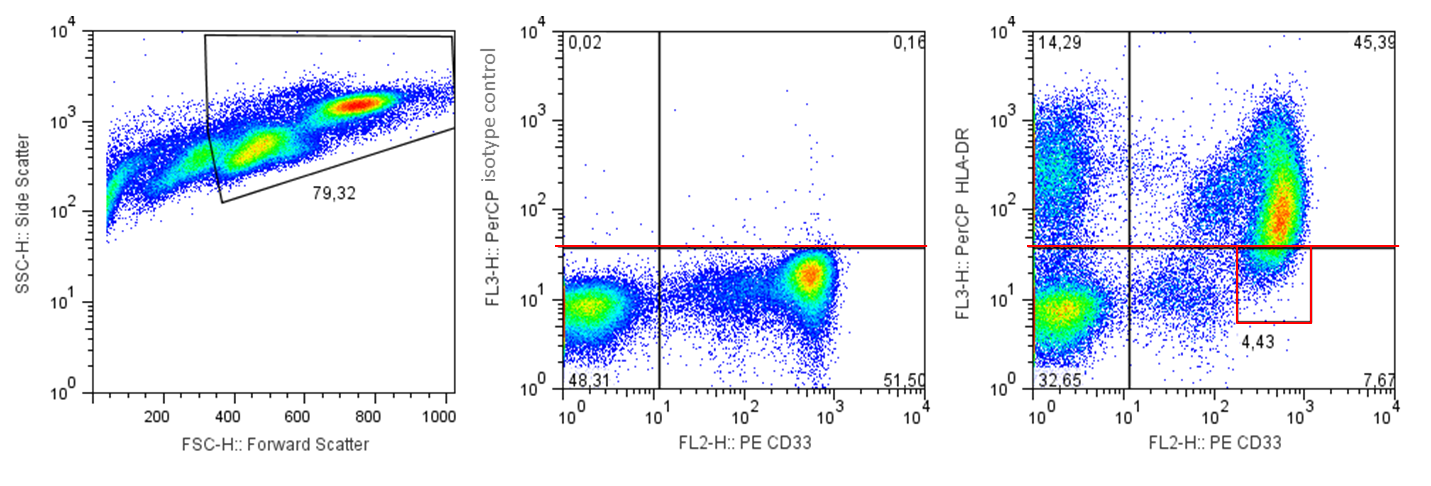
**

Representative dot blots and gating strategies for mMDSC. The first gate (black gate, first panel) is placed on the lymphocyte and monocyte fraction in FSC and SSC. Second panel shows PerCP isotype control (red line, second panel) which defines the HLA-DR negative population. In the third panel (red square gate, third panel) cells are HLA-DR^low/-^ and CD33^+^ and are defined as mMDSC. Gating was performed according to Dumitru et al. (Cancer Immunol Immunother 2013).
