# Supplementary material for: Kinetics of human myeloid-derived suppressor cells after blood draw
Source: J Transl Med. 2016 Jan 6;14:2. doi: 10.1186/s12967-015-0755-y (PMC4702395; doi:10.1186/s12967-015-0755-y)
Supplement: Supplementary file 2 — 10.1186/s12967-015-0755-y Gating of CD15+/CD66b+ population. [file 12967_2015_755_MOESM2_ESM.docx]

**Figure S2: Gating of CD15^+^/CD66b^+^ population**

**
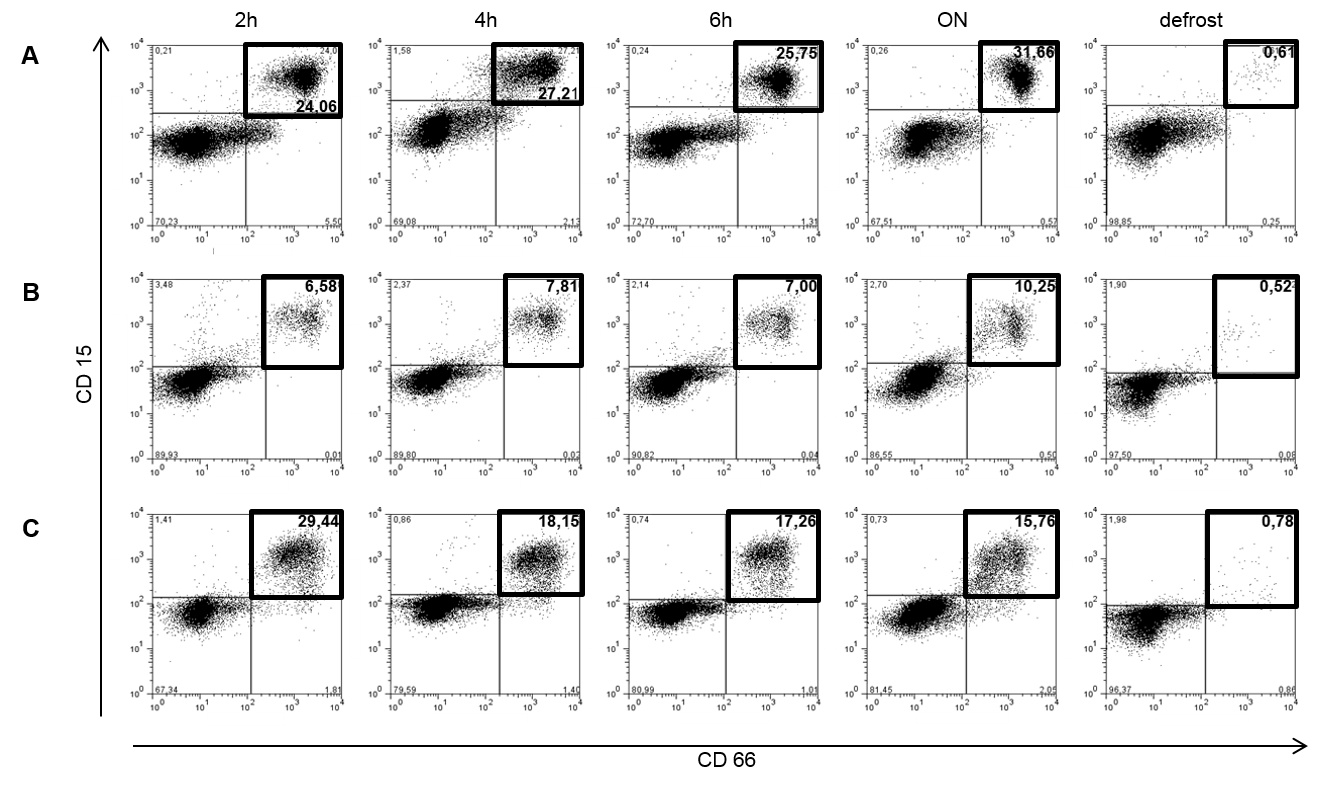
**

Representative dot plots of gMDSC of 3 HIV patients gated on CD15^+^/CD66b^+^ (rectangle gate).

Comparable to the CD11b^+^/CD14^-^ population, the majority of the CD15^+^/CD66b^+^ remained similar after 2h, 4h and 6h with a slight increase after ON (A and B). Again, the population disappeared after freezing/thawing procedure. The third panel (C) shows a minor divergent presentation with decreased gMDSC frequencies after 4h, 6h and ON compared to 2h time point.
